# Asian Cohort for Alzheimer's Disease (ACAD) Pilot StudyFocusing on Implementation Among Korean Americans

**DOI:** 10.1002/alz70858_106614

**Published:** 2025-12-26

**Authors:** Haeok Lee, Hyun‐Sik Yang, Hoowon Kim, Nayoung Kim, Yeunjoo E. Song, Younhee Kang, Kyungmin Kim, Eun Hyun Seo, Yong Jeong, Kun Ho Lee, Gyungah R Jun, Yun‐Beom Choi, The Asian Cohort for Alzheimer's Disease Study

**Affiliations:** ^1^ Meyers College of Nursing, New York University, New York, NY, USA; ^2^ Harvard Medical School, Boston, MA, USA; ^3^ Department of Neurology, Harvard Medical School, Boston, MA, USA; ^4^ Brigham and Women's Hospital, Boston, MA, USA; ^5^ Department of Neurology, School of Medicine, Chosun University/Chosun University Hospital, Gwangju, Korea, Republic of (South); ^6^ National Research Center for Dementia, Chosun University, Gwangju, Korea, Republic of (South); ^7^ Meyers College of Nursing, New York University, NY, NY, USA; ^8^ Department of Population and Quantitative Health Sciences, Institute for Computational Biology, Case Western Reserve University, Cleveland, OH, USA; ^9^ College of Nursing, Ewha Womans University, Seoul, Korea, Republic of (South); ^10^ Department of Child Development and Family Studies, Seoul, Seoul, Korea, Republic of (South); ^11^ Premedical Science, College of Medicine, Chosun University, Gwangju, Korea, Republic of (South); ^12^ Gwangju Alzheimer's and Related Dementia (GARD) Cohort Research Center, Chosun University, Gwangju, Korea, Republic of (South); ^13^ Department of Bio and Engineering, KAIST, Daejeon, Korea, Republic of (South); ^14^ Graduate School of Medical Science & Engineering, KAIST, Daejeon, Korea, Republic of (South); ^15^ Department of Life Sciences, Chosun University, Gwangju, Korea, Republic of (South); ^16^ Department of Biostatistics, Boston University School of Public Health, Boston, MA, USA; ^17^ Department of Medicine (Biomedical Genetics), Boston University Chobanian & Avedisian School of Medicine, Boston, MA, USA; ^18^ Department of Neurology, Rutgers New Jersey Medical School, Newark, NJ & Englewood Health, Englewood, NJ, USA; ^19^ Englewood Health, Englewood, NJ, USA

## Abstract

**Background:**

Alzheimer's disease is the leading cause of dementia and is rapidly increasing among the Asian American population. The Asian Cohort for Alzheimer's Disease (ACAD) was launched to address the underrepresentation of Asian American subjects in clinical research. The purpose of this report is to describe and evaluate the feasibility of a culturally informed and community‐engaged genetic‐clinical study.

**Methods:**

Older Korean American adults were recruited and cognitive, early lifestyles, and neurological assessment data were collected along with biological samples. A purposeful sampling method was utilized, and bilingual Korean registered nurses and neurologists collected the data. Consensus meetings were held to review each case and to make a diagnosis of normal control (NC), subjective cognitive complaints (SCC), mild cognitive impairment (MCI), and dementia.

**Results:**

A total of 60 participants were recruited during the 12‐month period from 2020 to 2021, meeting the targeted number of participants. The mean age of the participants was 79.58 (±8.05) with a range of 60‐101. Seventy percent (70%) of participants were female and 80% had at least high school education. The length of stay in the U.S. was 35.23 (±13.36) years, 66.67% had limited English proficiency, and 16.67% lived alone. 67.67%, 63.33%, and 43.33% of participants reported having hypertension, high cholesterol and diabetes, respectively. Fifty‐four participants completed cognitive assessment batteries and neurological assessments, and the biological samples were collected from 49 participants (33 blood and 16 saliva). Six participants who missed the neurological exams either relocated to live closer to their relatives or forgot the data collection appointment. Among 54 participants, 22 (36.67%) were NC and 32 participants had some cognitive deficit (25% dementia, 10% MCI, and 18.33% SCC).

**Conclusions:**

Successful implementation of an ACAD study depends on several factors including close collaboration with participants, community recruiters, data collectors, clinicians, and biological sample handling to ensure that any data collection times and locations are participant friendly. This pilot study offered new insight into Alzheimer's disease and related dementia studies with Korean American older adults including the feasibility of community engaged recruitment and data collection on cognition, lifestyle, neurological examination, biological sampling, and lessons learned from this study.

